# 3D-Printed PLA Scaffold with Fibronectin Enhances In Vitro Osteogenesis

**DOI:** 10.3390/polym15122619

**Published:** 2023-06-08

**Authors:** Eisner Salamanca, Cheuk Sing Choy, Lwin Moe Aung, Ting-Chia Tsao, Pin-Han Wang, Wei-An Lin, Yi-Fan Wu, Wei-Jen Chang

**Affiliations:** 1 School of Dentistry, College of Oral Medicine, Taipei Medical University, Taipei 110301, Taiwan; eisnergab@tmu.edu.tw (E.S.); d204111006@tmu.edu.tw (L.M.A.); a0958237236@gmail.com (T.-C.T.); b202108012@tmu.edu.tw (P.-H.W.); b202108027@tmu.edu.tw (W.-A.L.); 2 Department of Community Medicine, En Chu Kong Hospital, New Taipei City 237, Taiwan; 01610@km.eck.org.tw; 3 Department of Nursing, Yuanpei University of Medical Technology, Hsinchu 300, Taiwan; 4 Dental Department, Taipei Medical University, Shuang-Ho Hospital, Taipei 235041, Taiwan

**Keywords:** polylactic acid, fibronectin, fused deposition modeling, osteogenesis, biocompatibility

## Abstract

Background: Tricalcium phosphate (TCP, Molecular formula: Ca_3_(PO_4_)_2_) is a hydrophilic bone graft biomaterial extensively used for guided bone regeneration (GBR). However, few studies have investigated 3D-printed polylactic acid (PLA) combined with the osteo-inductive molecule fibronectin (FN) for enhanced osteoblast performance in vitro, and specialized bone defect treatments. Aim: This study evaluated PLA properties and efficacy following glow discharge plasma (GDP) treatment and FN sputtering for fused deposition modeling (FDM) 3D printed PLA alloplastic bone grafts. Methods: 3D trabecular bone scaffolds (8 × 1 mm) were printed by the 3D printer (XYZ printing, Inc. 3D printer da Vinci Jr. 1.0 3-in-1). After printing PLA scaffolds, additional groups for FN grafting were continually prepared with GDP treatment. Material characterization and biocompatibility evaluations were investigated at 1, 3 and 5 days. Results: SEM images showed the human bone mimicking patterns, and EDS illustrated the increased C and O after fibronectin grafting, XPS and FTIR results together confirmed the presence of FN within PLA material. Degradation increased after 150 days due to FN presence. 3D immunofluorescence at 24 h demonstrated better cell spreading, and MTT assay results showed the highest proliferation with PLA and FN (*p* < 0.001). Cells cultured on the materials exhibited similar alkaline phosphatase (ALP) production. Relative quantitative polymerase chain reaction (qPCR) at 1 and 5 days revealed a mixed osteoblast gene expression pattern. Conclusion: In vitro observations over a period of five days, it was clear that PLA/FN 3D-printed alloplastic bone graft was more favorable for osteogenesis than PLA alone, thereby demonstrating great potential for applications in customized bone regeneration.

## 1. Introduction

Bone resorption after tooth extraction is a significant concern due to its impact on the patient’s oral health. It has been proved that two-thirds of bone loss can occur within the first three months after extraction [1], which can leave the edentulous area without the adequate bone volume for successful future implant treatment. To reconstruct the bone loss, various treatments have been developed, including guided bone regeneration (GBR), which uses bone grafting and prevent further loss by filling and remodeling the dental bone defects.

Alloplastic bone grafts have been developed to closely mimic the biological properties of natural bone, such as osteoconductivity and osteointegration. For example, calcium phosphate ceramics (e.g., hydroxyapatite (HA)), tricalcium phosphate (TCP), decellularized bone matrix (DCB), polymers (e.g., polymethylmethacrylate (PMMA)), and metals (e.g., nickel-titanium), which are commonly used synthetic bone substitute materials used in dental and orthopedic treatments.

Polylactic Acid (PLA) is a promising biodegradable polymer with favorable hydrophilic surface properties and biocompatibility. It also has excellent mechanical properties such as high tensile strength (>40 MPa), high Young’s modulus (>1000 MPa), and low elongation at break (<15%), making it an ideal candidate for bone tissue engineering. Glow discharge plasma (GDP) treatment is an effective and well-established method for surface modification of biomaterials. This treatment can be used to remove impurities and deposit functional proteins on the surface of synthetic bone substitutes [2,3,4].

Fibronectin (FN) is a high molecular weight glycoprotein (440~500 kDa) and a major component of the extracellular matrix (ECM). FN is known to exhibit significant osteoinductive effects on the proliferation of mesenchymal stem and endothelial cells [5]. Recently, novel preparation techniques and strategies using GDP for synthetic bone substitutes have been selected to improve the scaffold’s properties efficiently. For instance, incorporating FN can enhance synthetic bone substitutes and promote better guided bone tissue regeneration [6,7].

In recent years, 3D printing technology has gained attention for creating bone graft substitutes by modifying the physical properties and structure of synthetic bone substitutes, enabling a perfect fit of bone graft material to the bony defect prior to even the patient’s surgery. This can reduce surgery time, prevent contamination of the alloplastic graft material with oral bacteria, and more importantly, improve the outcomes of GBR treatment, resulting in faster and more efficient osteogenesis. Fused Deposition Modeling (FDM) is a 3D printing method that uses filament-shaped thermoplastic polymers as construction material. The filament’s material is heated, melted in the printing head, and extruded onto the build plate, depositing layers to form the desired structure.

Due to its bio-compatibility and mechanical strength, PLA has been widely used in various surgical implant materials such as pins, screws, plates, and wires [8]. Several recent studies have incorporated polymer matrix with calcium phosphate ceramics to fabricate 3D printed biomedical scaffolds for promoting both bone regeneration and angiogenesis [9,10]. Evidence suggests that an optimal composite biomaterial can be achieved by incorporating an osteoconductive filler with varying powder into PLA, with the filler content ideally mass ranging from 10 to 30% [11,12]. The mixture ratio of pure biomaterials can be adjusted to achieve optimal absorption and improve mechanical properties, in order to enhance osteogenesis and reduce bone destruction in the bone defects [13]. Hence, this study aimed to explore the potential of 3D-printed PLA alloplastic bone biomaterials, with a particular emphasis on grafting the osteo-inductive FN molecule onto the scaffold. We aimed to graft these biomaterials with an osteo-inductive FN molecule and utilize FDM technology to create more personalized alloplastic bone grafts that mimic human bone structure.

## 2. Results

### 2.1. Scanning Electron Microscopy and Energy Dispersive Spectrometry Testing

**Figure 1:**
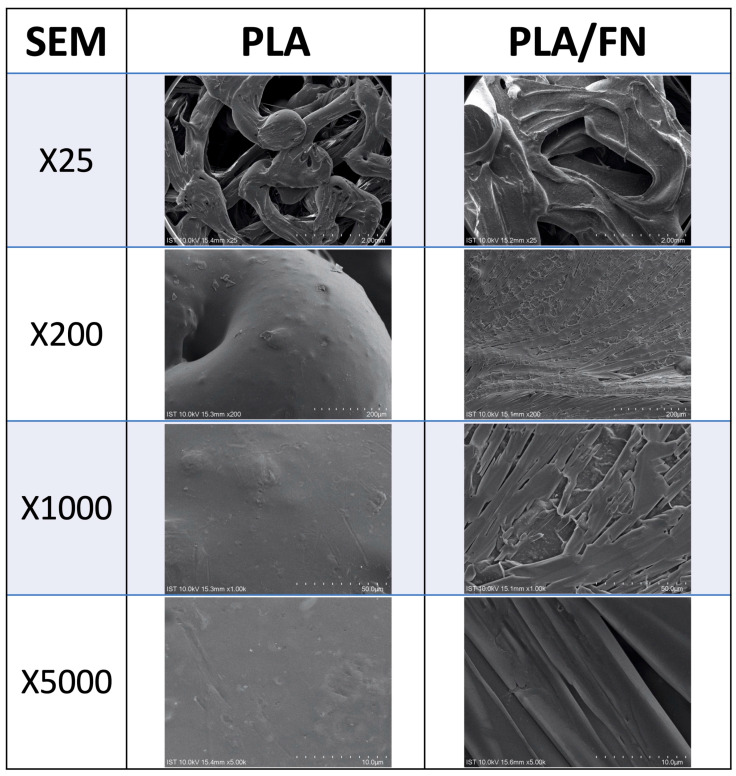
SEM micrographs of different 3D printed samples mimicking human bone patterns and exhibiting similar surface roughness (25× and 5000×).

The surface morphology at the macro scale (25× and 200× images) displayed similar surface roughness and patterns in all 3D printed samples (Figure 1). On a micro scale, PLA and PLA/FN rough surfaces were be observed (5000× Figure 1).

Quantitative topographical evaluations conducted by EDX revealed that all samples contained oxygen, and carbon (Table 1). The weight percentages of Carbon, and Oxygen in PLA were 51 ± 1.26% and 42.93 ± 2.50%, respectively. The weight percentage of these PLA elements weights increased in the PLA/FN samples at 57 ± 3.21% Carbon and 47.54 ± 3.21% Oxygen, due to FN adhesion on the surfaces, while maintaining the same Calcium weight percentage % (Table 1).

### 2.2. Photoelectron Spectrometer Element Analysis

The XPS elemental atomic percentage results are listed in Table 2, showing the identified elements: C1s, N1s, O1s, Mg2s, Ca2p, and Na1s. C1s exhibited a similar tendency as the EDX results, indicating an increase due to FN adhesion on the surfaces. A decrease in O1s and N1s in the PLA/FN was observed, resulting from FN coating on these 3D-printed samples.

### 2.3. Functional Group Result by ATR-FTIR

**Figure 2:**
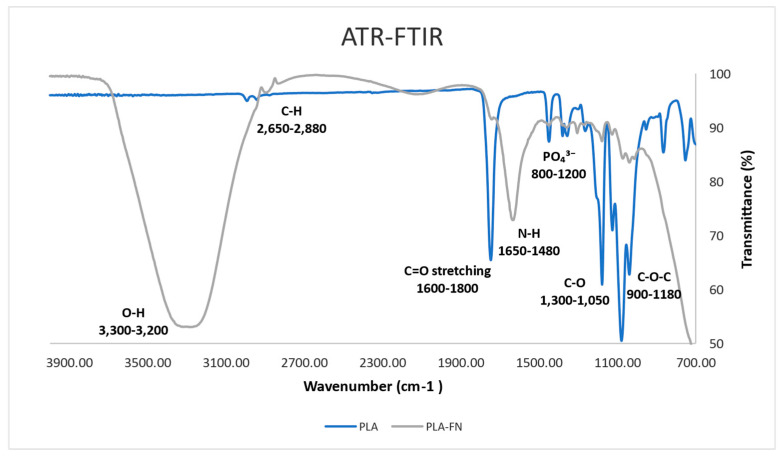
Analysis of atomic peaks and functional groups via infrared spectroscopy (FTIR) spectra.

As depicted in Figure 2, C–O–C (Carboxyl stretching) was present in the absorption band at 900–1180 cm^−1^. For both groups, the absence of a band around 3500 stretches (O–H grouping) can be observed. Multiple peaks between 800 and 1200 cm^−1^ signify the presence of C–O and PO_4_^3−^ ions. C–O, N–H, C–H, and O–H functional bonds were detected in samples treated with fibronectin, by absorption band maxima at 1050, 1620, 2700, and 3200 cm^−1^.

### 2.4. Degradation

**Figure 3:**
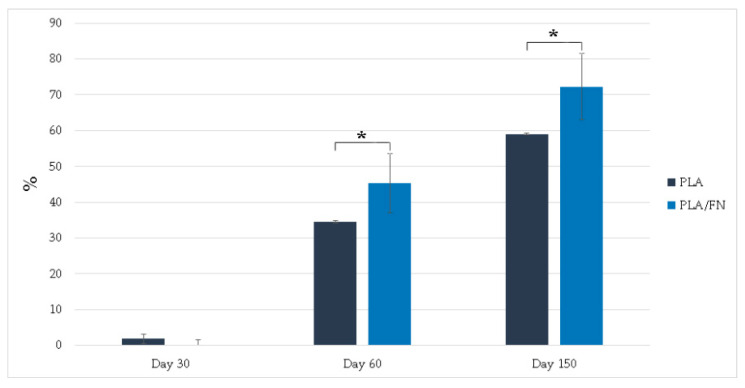
Degradation rate increased with the presence of FN in PLA scaffold. * *p* < 0.05, significant difference.

As shown in Figure 3, after sixty days of testing same tendency in degradation for all materials was observed up to 150 days. At this time point, the PLA/FN 3D printed samples showed a 72.26 ± 9.22% degradation, which was better than pure PLA (59 ± 0.19%), this can be attributed to the presence of FN (Figure 3).

### 2.5. Cell Viability and Proliferation

**Figure 4:**
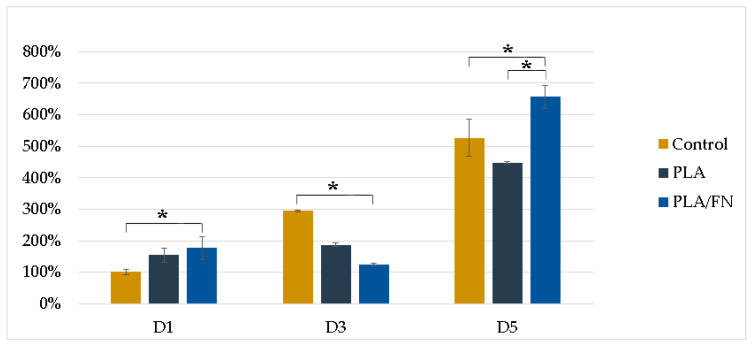
Proliferation determined by MTT cells assay of MG-63 on different 3D printed samples. * *p* < 0.05, significant difference.

**Figure 5:**
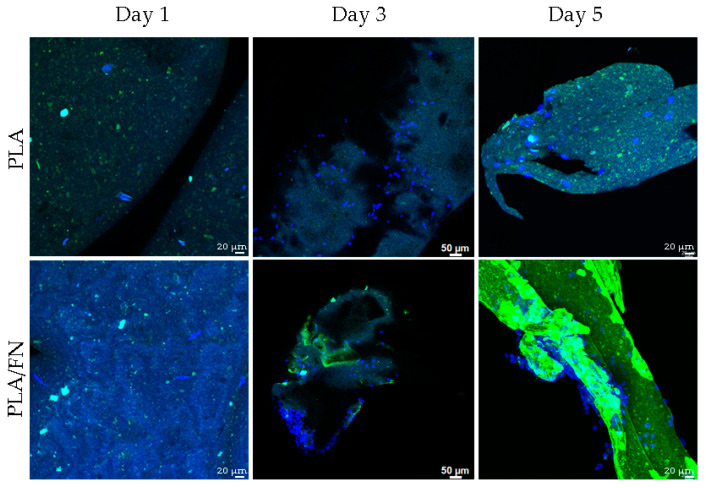
24-h cell culture with DAPI/phalloidin immunofluorescent imaging, indicating similar attachment on PLA and slightly better spreading on 3D printed PLA/FN.

MTT assay and 3D culture immunofluorescence (DAPI-Phalloidin) were utilized to determine cell viability and proliferation. PLA/FN demonstrated the highest viability and proliferation on days 1 and 5, with 177.30% and 656.65% over control, respectively. This indicates that PLA/FN 3D printed biomaterial was the best option for cell proliferation (Figure 4). Phalloidin/DAPI immunofluorescence staining revealed the 3D culture of these cells on PLA/FN at 1, 3, and 5 days, proliferating and exhibiting spindle shapes with filopodial extensions, which indicates higher cell attachment on the biomaterial surface (Figure 5).

### 2.6. Alkaline Phosphatase Activity

**Figure 6:**
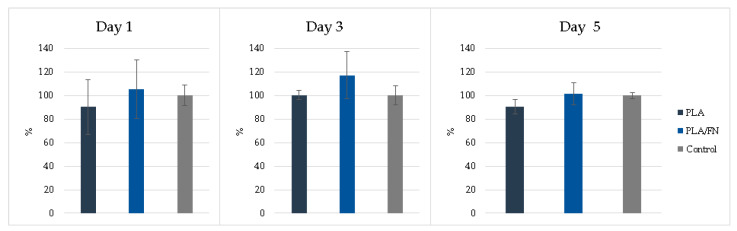
Alkaline phosphates activity detection was similar for all 3D printed biomaterials, with PLA/FN exhibiting a slightly higher percentage than other groups.

Alkaline phosphatase activity detection showed similar results without any significant difference for all 3D-printed synthetic bone alloplast samples on all days (Figure 5). PLA/FN had a slightly better ALP percentage compared to the other groups and resembled ALP gene expression results at day 5 (Figure 5 and Figure 6).

### 2.7. Real-Time PCR

**Figure 7:**
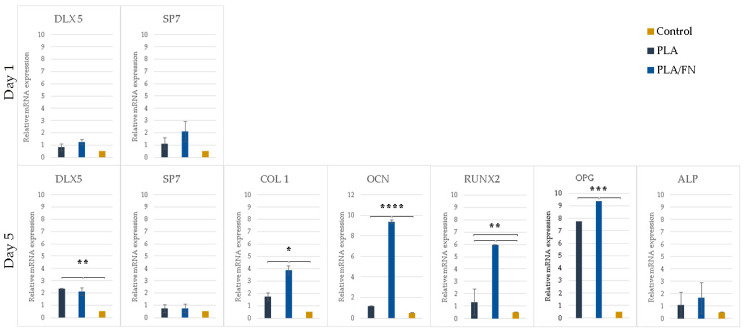
mRNA expression of osteogenesis related genes. PLA/FN 3D printed samples showed the highest gene expression. * *p* < 0.05, ** *p* < 0.01, *** *p* <s 0.001, **** *p* < 0.001 Significant differences.

The expression of osteogenesis-related genes such as OCN, RUNX2, ALP, and COL 1 were not expressed on day 1 but showed upregulation in PLA/FN on day 5. Sp7 is an essential transcription factor for *osteoblast* differentiation, but cells cultivated in all the 3D printed biomaterials had similar gene expression on days 1 and 5, without any statistically significant difference. The expression of immature osteoblasts maker, RUNX2, was absent on day 1, and later increased exponentially in cells cultured with PLA/FN (*p* < 0.01). The positive regulator for osteoblastogenesis, DLX5, was upregulated in cells cultured with PLA and PLA/FN (*p* < 0.01) compared to the other 3D-printed samples. OPG, an osteoclast differentiation factor, had the highest expression in cells cultured with PLA/FN (*p* < 0.001). Overall, cells cultured with PLA/FN demonstrated the best expression of genes related to osteoblast-like cells and osteogenesis (Figure 7).

## 3. Discussion

As a promising approach for bone defect regeneration, 3D printing technology offers the ability to control the internal microstructure and geometry of synthesized matrixes matrices [14]. Further research on the appropriate design and material properties, which should be tailored to scaffolds according to different types of bone defects and fracture sites, is required to develop more clinically practical scaffolds. A material should first and foremost be biocompatible, interacting with living cells and tissues without causing undesirable physiological responses [15]. Customized bone repair biomaterials and their fabrication are still being investigated. Three-dimensional (3D) printing is a high-speed fabrication process for bone tissue biomaterials that paves the way for novel approaches to clinical bone defect problems [16]. Synthetic bone substitutes can be 3D printed to create a perfect fit between the bone graft material and the bony defect before even starting the patient’s surgery. This can improve the time needed for surgery, avoid contamination of the alloplast graft material with oral bacteria, and more importantly, enhance the outcomes of guided bone regeneration treatment, resulting in faster and more osteogenesis. Therefore, the aim of this study was to investigate 3D printed PLA alloplast bone biomaterials and graft the osteoinductive FN molecule to it, and later 3D print it with FDM for a more personalized alloplast bone graft.

FDM commonly uses biocompatible polymers with low melting temperatures such as PCL, poly(lactic acid) (PLA), and PLGA, which are frequently combined to create tissue-engineered scaffolds [17]. Many studies [18,19,20] have shown that these FDM-based scaffolds have favorable mechanical and biochemical properties for bone regeneration, similar to the findings of the present study; the surface morphology at macro scale (25× and 200× images) showed similar surface roughness and patterns in all 3D printed samples (Figure 1). The quantitative topographical evaluations performed by EDX revealed all samples contained Oxygen, and Carbon (Table 1); this was supported by the XPS results listed in Table 2, showing the elements identified as C1s, N1s, O1s, Mg2s, Ca2p, and Na1s. C1s exhibited a similar tendency to the EDX results, indicating an increase due to FN adhesion on the surfaces. As shown in Figure 2, after sixty days of testing, it was possible to observe almost half of the degradation in PLA/FN 3D printed samples, which was slightly more than in pure PLA; this can be attributed to the presence of FN. No functional group was observed around 3500 stretches demonstrating the absence of byproducts of hydrolysis of PLA [21]. Successful incorporation of β-TCP into PLA can be confirmed by FTIR peaks between 800–1200 cm^−1^ and presence of COO–H and N–H functional groups at 1050, 1620, 2700 and 3200 cm^−1^ verified the attachment of fibronectin proteins on the samples.

Another study previously found that PLA scaffolds with different pore sizes, produced by FDM, had reasonable distributions of human bone marrow stromal cells (hBMSCs) [22]; the present study used different cells but also observed similar cell distribution on the surface of the 3D printed biomaterial. In another study, MG63 cells were regularly shaped and displayed spreading filopodia connected to the sample surface [23] on day one, similar to the cell culture results with DAPI/phalloidin immunofluorescent imaging in the present study at 1, 3, and 5 days (Figure 4).

PLA/FN 3D printed biomaterial proved to be the best option for cell viability and proliferation (Figure 3 and Figure 4). However, all cells cultured with the 3D printed synthetic human bone pattern had similar alkaline phosphatase activity without any statistically significant difference (Figure 5). This was supported by a similar ALP gene expression trend at 5 days after culture. Despite this similarity, the expression of osteogenesis-related genes like OCN, RUNX2, and COL exhibited higher expression at day 5 in PLA/FN cultured cells compared to PLA and control. Sp7 is an essential transcription factor for *osteoblast* differentiation, but cells cultivated in all the 3D printed biomaterials had similar gene expression at day 1 and 5 without any statistically significant difference. The expression of immature osteoblasts, RUNX2, was nonexistent on day 1 and later increased exponentially in cells cultivated with PLA/FN (*p* < 0.01). The positive regulator for osteoblastogenesis, DLX5, was upregulated in cells cultivated with PLA and PLA/FN (*p* < 0.01) over the 3D-printed samples. OPG osteoclast differentiation factor had the highest expression in cells cultivated with PLA/FN (*p* < 0.001). Overall, cells cultivated with PLA/FN (Figure 6) had the best expression of genes related to osteoblast-like cells and promoted osteogenic differentiation of MG-63 cells in vitro. Similar osteogenic differentiation in vitro has been described with FDM 3D printing, but using polycaprolactone (PCL) and zinc. To the best of our knowledge, this is the first time fibronectin has been used successfully to enhance 3D printed PLA human bone patterns [24]. However, there are still some limitations in our study. Attachment affinity and bonding of fibronectin proteins on PLA material surface are unclear and the details mechanism of osteo-inductive effects on PLA is still unknown. Further osseointegration properties needed to be found out more in in vivo study to confirm the data obtained in this study. Additionally, it is still unknown whether the material will retain its osteo-inductive properties after mixing PLA with more osteoconductive materials, such as beta Tricalcium phosphate or hydroxyapatite (HA). Due to the limitations of the study, more in vitro and in vivo studies are necessary to utilize this approach for bone defect treatments.

## 4. Methods

### 4.1. Scaffold Preparation

#### 4.1.1. D-Printed Trabecular Bone Scaffold Manufacturing

Based on previous optimal findings [25], 100% PLA (molecular weight: 60,000 Mn, 38534-1G Sigma-Aldrich, MO, USA). The mixing process began by pouring PLA and β-TCP materials into a single screw extruder (single screw mixer; Filabot EX2 and spooler; Filabot, Barre, VT, USA) equipped with an 8-mm diameter circular aperture for mixing and extrusion.

**Figure 8:**
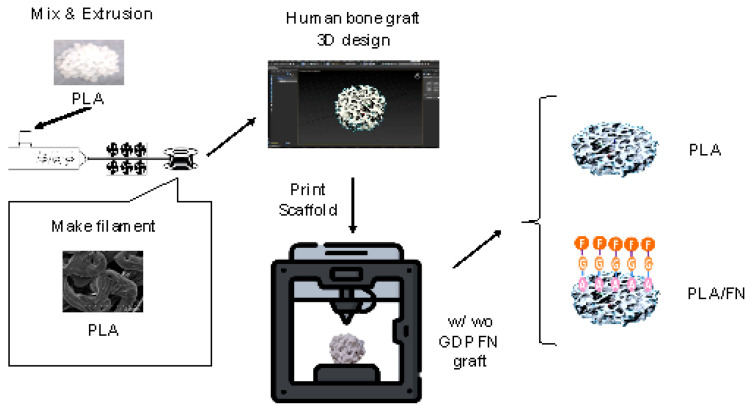
The prototype of 3D trabecular bone scaffold in 3D Max software and its 3D printed version. Scale bar = 8 mm.

The 3D trabecular bone scaffold model for printing was created by using the human cancellous bone pattern taken from a computerized tomography in 3D Max software, as shown in Figure 8. This scaffold (8-mm diameter and 1-mm height) was printed by the 3D printer (XYZ printing, Inc. 3D printer da Vinci Jr. 1.0 3-in-1). In this study, we used a human CT scan as the template for all samples. The samples, printed out through a 3D printer, mimicked the human cancellous bone’s pore size of 200–500 μm and porosity of 75–85%. The printer was equipped with a 0.4 mm wide nozzle head moving at a speed of 400 mm/s, and it utilized a 1.75 mm diameter from both PLA/FN and PLA filaments at 210 °C. The layer resolution ranged from 100 to 400 μm for a high fidelity of the 3D printed human bone structure.

#### 4.1.2. GDP Treatment and FN Grafting

For FN grafting, GDP was used to clean PLA samples with argon gas through the glass tube (PJ; AST Products Inc., North Bellerica, MA, USA) with 85-W argon gas and a radiofrequency of 13.56 MHz at room temperature for 10 min. The scaffold surface’s impurities were removed during this argon plasma treatment. Next, NH groups were attached to the two argon-treated scaffolds in a GDP machine filled with allylamine (AA) gas. The scaffolds were subjected to AA gas in the GDP reactor at room temperature with 85W [3,4,26], 13.56 MHz, and 100 millitorrs (mTorr) for 30 min. The scaffolds coated with AA were called PLA/FN. Then, these samples were removed from the plasma chamber and immediately immersed in a 3% glutaraldehyde (GA) solution (Merck, NJ, USA) for 30 min. The GA solution was used to link amine groups (-NH_2_) on the surface of the scaffold discs. After two rounds of rinsing with 0.1 M phosphate-buffered saline (PBS, Wako Pure Chemical Industries, Osaka, Japan), the scaffold discs were immersed in a 5 μg/mL FN solution (Sigma-Aldrich Co., St. Louis, MO, USA) for 24 h to graft the surface with FN. Finally, the 3D printed scaffold discs were submerged in a Tris-phosphate buffer (2-amino-2-hydroxymethyl-1,3-propanediol 999, Wako Pure Chemical Industries, Osaka, Japan, pH 7.4) solution for 30 min to break the chain reaction between AA and FN.

### 4.2. Material Characterization

#### 4.2.1. Scanning Electron Microscopy (SEM) and Energy Dispersive Spectrometry (EDS)

A layer of gold film was coated on the surfaces of the scaffold specimens (control and test groups sample size, n = 3). The observed surface area of different scaffolds was chosen for photographs, which operate at 15 KV with 25×, 50×, 250×, 1000×, and 5000× magnification. Images were taken from at least five random, nonoverlapping flat areas. Microphotography was taken with a scanning electron microscope (SEM S-2400; Hitachi, Ltd., Tokyo, Japan), and the composition of element analysis was determined by energy dispersive spectroscopy (EDS, Bruker Quantax EDS, Berlin, Germany).

#### 4.2.2. X-ray Photoelectron Spectroscopy (XPS)

X-ray photoelectron spectroscopy (XPS, Perkin-Elmer Phi ESCA 5500 system) was employed to identified the elements and their chemical bonds on the surface of the 3D-printed samples using depth profiling (control and test groups sample size, n = 3). The system was equipped with a monochromated 450 W Al Kα source. XPS is based on the photoelectric effect principle. During the measurement, a 220 W source power and an angular acceptance of take-off angle of 15° were used [27]. Core levels of C1s, N1s, O1s, Mg2s, Ca2p, and Na1s core levels were recorded. Each sample was analyzed at least 3 times.

#### 4.2.3. Functional Group Analysis by ATR-FTIR

ATR-FTIR analysis of 3D printed samples (control and test groups sample size, n = 3) were performed by using a Nicolet iS5 (Thermo Fisher Scientific, Madison, WI, USA) equipped with an iD7 crystal ZnSe in reflection mode. The absorbance spectra of control and fibronectin-grafted samples were measured using 16 scans with a resolution of 0.482 cm^−1^. We obtained FITR spectra in the wavenumber range 4000–650 cm^−1^ and used a background spectrum to normalize the spectra. Absorbance of spectra is measured in order to derive atomic peaks.

#### 4.2.4. Degradation

Degradability test was conducted by immersing the dried samples in a phosphate-buffered saline solution (PBS, pH 7.4) at 25 °C, following mass-to-volume ratio of 1 g: 15 mL based on GB/T 16886 series standards [28]. The samples (control and test groups sample size, n = 3) were rinsed in distilled water and dried at each soaking time (Days 30, 60, and 150). After drying, the samples were weighed using an electric balance accurately (Accuracy: 0.0001 g, Sartorius, BSA2245-CW, China). The degradation rate was reported as the weight loss, according to the calculated formula: Weight loss rate (%) = (M_0_ − Mt)/M_0_ × 100%, where M_0_ and Mt represent the sample weights before and after incubation in PBS, respectively. The composition of phosphate buffer saline (PBS) was 1.37 M NaCl, 27 mM KCl, 18 mM KH_2_PO_4_, and 100 mM NaH_2_PO_4_. It was diluted with deionized water (ddH_2_O) at a ratio of 1:9. 100 mL of the original PBS solution was mixed with 900 mL of ddH_2_O, resulting in a volume of 1000 mL.

### 4.3. Biocompatibility Evaluation

#### 4.3.1. Cell Culture and Seeding

MG-63 human osteoblast-like cells were purchased from the Bioresource Collection and Research Center (BCRC, Hsinchu, Taiwan). Following a published protocol, MG-63 cells were cultured in Dulbecco’s Modified Eagle’s Medium (DMEM; HyClone, Logan, UT, USA) supplemented with L-glutamine (4 mmol/L), 10% fetal bovine serum, (DMEM; HyClone, Logan, UT, USA) at 37 °C in a humidified atmosphere serum, and 1% penicillin-streptomycin (HyClone, Logan, UT, USA) at 37 °C in a humidified atmosphere containing 95% air and 5% CO_2_. Upon reaching approximately 80% confluence, cell concentration was adjusted to 1 × 10^4^ cells/mL and seeded into 24-well Petri dishes (Costar Corporation, Cambridge, MA, USA) for subsequent experiments.

#### 4.3.2. Cell Viability

Cell viability was assessed using a 3-4,5-dimethylthiazol-2-yl)-2,5-diphenyltetrazolium bromide (MTT) reduction assay (Roche Applied Science, Mannheim, Germany). After MTT salt was added, formazan was produced in living cells by mitochondrial dehydrogenase, according to the manufacturer’s instructions. Formazan dye was solubilized with dimethyl sulfoxide (DMSO) for 10 min, causing a color change from yellow to dark blue. Subsequently, the optical density of the medium was quantified using an ELISA reader (SpectraMax iD3 Multi-Mode Detection Platform, Molecular Devices, USA) at 570 nm. Cell viability was expressed as a percentage, assigning the 100% optical density value to the absorbance of the control cells. In this study, each group was repeated at least 3 times and evaluated on days 1, 3, and 5.

#### 4.3.3. Immunofluorescence

After 24 h of MG-63 cell growth, all samples were removed from their medium, rinsed with PBS (10 mM, pH 7.4), and fixed with 4% paraformaldehyde (PFA) at room temperature. Cells were permeabilized with 1% Triton X-100 in PBS for 10 min at room temperature. After three washes with 0.1% Triton X-100 in DPBS, nuclei were stained for 1 h with DAPI (1:1000 dilution; 5 mg/mL stock solution; Sigma-Aldrich) and Alexa Fluor 488 Phalloidin (1:80 dilution; catalog # A12379; Invitrogen). PBS was used to eliminate excess coloring (10 mM, pH 7.4) [29]. Leica STELLARIS 8 systems were utilized to evaluate the dispersion of cells throughout the various 3D-printed samples (control and test groups sample size, n = 3).

#### 4.3.4. Alkaline Phosphatase (ALP) Activity

ALP level was determined in MG-63 (1 × 10^4^ cells/mL). Each sample was evaluated on day 1, 3, and 5 after medium substitution with each 3D printed sample (control and test groups sample size, n = 3). According to the manufacture’s protocol (BioVision, ALP kit catalog #K412-500, Milpitas, CA, USA), intracellular ALP measurement was performed by putting the culture supernatants in 100 µL of assay buffer in a 96-well plate (Costar Corp., Cambridge, MA, USA). Subsequently, 20 µL of stop solution and 50 µL of 5 mM 4-Nitrophenyl Phosphate (pNPP, Merck, Schuchardt, Hohenbrunn bei München, Germany) were added to each well. After pipetting and waiting for hydrolyzing by ALP in 60 min at 25 °C, yellow product (nitrophenol) formed later. An enzyme-linked immunosorbent assay reader (SpectraMax iD3 Multi-Mode Detection Platform, Molecular Devices, San Jose, CA, USA) was used to evaluate the absorbance, which will be measured at a wavelength of 405 nm, using 620 nm as the reference. The activity of ALP (unit/L) was determined.

#### 4.3.5. Relative Quantitative Real-Time Polymerase Chain Reaction (qPCR)

Quantification of all gene transcripts was performed at 1, 3, and 5 days after cell culture and seeding (control and test groups sample size, n = 3). Total RNA extraction and purification were carried out using the Novel Total RNA Mini Kit (NovelGene, Molecular Biotech, New Taipei, Taiwan), according to the manufacturer’s instructions. For RNA processing, cells were used trypsin, harvested, and resuspended in 100 μL of PBS before being subjected to cell lysis by adding 400 μL of natural rubber and 4 μL of S-mercaptoethanol to the sample. RNA binding was later performed with 400 μL of 70% ethanol and centrifuged at 13,000 rpm. Afterward, the sample was washed and eluted with 50 μL of RNase-free water [30]. Next, purified RNA was quantified using an ND-1000 spectrophotometer (Nanodrop Technology, Wilmington, DE, USA). Each sample was stored at −20 °C for analysis real-time polymerase chain reaction (qPCR).

The expression levels of osteoblast markers, including osteopontin (OCN), Distal-Less Homeobox 5 (DLX5), osteocalcin (OCN), Runt-related transcription factor 2 (RUNX2), transcription factor Sp7 (SP7), Alkaline phosphatase (ALP), Collagen type 1 (COL1), osteoprotegerin (OPG), and the receptor activator of nuclear factor kappa B ligand (RANKL) were quantified using qPCR. Gene expression levels were normalized to the expression of the housekeeping gene glyceraldehyde 3-phosphate dehydrogenase (GAPDH). The control cell’s genes were set as the calibrator sample in DEME medium, representing the transcript amount expressed on day 0 of cells cultured only in DMEM medium [31].

Real-time PCR was performed using 2 μL of cDNA a 20 μL of reaction volume with a LightCycler^®^ 96 Instrument, an application software (Roche Molecular Systems, Inc., California, USA), and the Fast SYBR^TM^ Green Master Mix (Thermo Fisher, Cat#4344463, Madison, WI, USA). The temperature profile of the reaction was 95 °C for 10 min, followed by 40 cycles of denaturation at 95 °C for 15 s, annealing at 60 °C for 60 s, and extension at 72 °C for 30 s. Quantification was performed using the delta-delta calculation method. The cycle threshold (*C*_T_) value was used as an indicator, and the gene expression levels were normalized by using GAPDH levels in each sample to account for differences in total RNA content in the individual samples. Forward and reverse primer sequences were designed using Primer-BLAST from the U.S. National Library of Medicine, as listed in [30].

## 5. Conclusions

Our study concludes that 3D-printed PLA/FN human bone pattern alloplasts demonstrated superior outcomes compared to PLA alone. This affirms our hypothesis that the incorporation of Fibronectin into PLA scaffolds enhances the quality of PLA in vitro, thus amplifying its potential for personalized osteogenesis treatments.

## Figures and Tables

**Table 1:** Weight ratio percentages of 3D printed samples.

Sample (Wt %)	C	O	Ca
PLA	51 ± 1.26	42.93 ± 2.50	0
PLA/FN	57 ± 3.21	47.54 ± 3.21	0

**Table 2:** Surface chemistry atomic percentages of 3D printed sample.

Sample	C1s	N1s	O1s	Mg2s	Si2p	Ca2p	Na1s	F1s	Cl2p
PLA	68.8	1.1	25	0	3.8	0.7	0	0	0
PLA/FN	72	3.6	22.1	0	1.2	0.2	0.2	0.9	0.2

## Data Availability

Data is contained within the article.
